# An impinging remnant meniscus causing early polyethylene failure in total knee arthroplasty: a case report

**DOI:** 10.1186/1752-1947-1-48

**Published:** 2007-07-13

**Authors:** Rachid Saouti, Barend J van Royen, Christiaan M Fortanier

**Affiliations:** 1 Department of Orthopaedic Surgery, VU University Medical Center, Amsterdam, The Netherlands

## Abstract

The management of patients with an apparently normal functional total knee arthroplasty (TKA) suffering from unexplained persistent pain and swelling is a challenging issue. The usual causes of pain after total knee replacement are well known, but there are a small number of patients in whom its aetiology is obscure. Malfunction due to soft tissue impingement has rarely been reported. A patient with an unusual case of posterior soft tissue impingement secondary to a trapped posterior horn of a remnant medial meniscus after TKA and responsible for severe early polyethylene wear, is reported. The diagnosis was confirmed by arthroscopy. Treatment was performed by arthrotomy. The meniscus remnant was removed followed by total synovectomy and isolated exchange of the polyethylene insert. To our knowledge, this is the first well-documented case reporting this association.

## Case presentation

**Figure 1:**
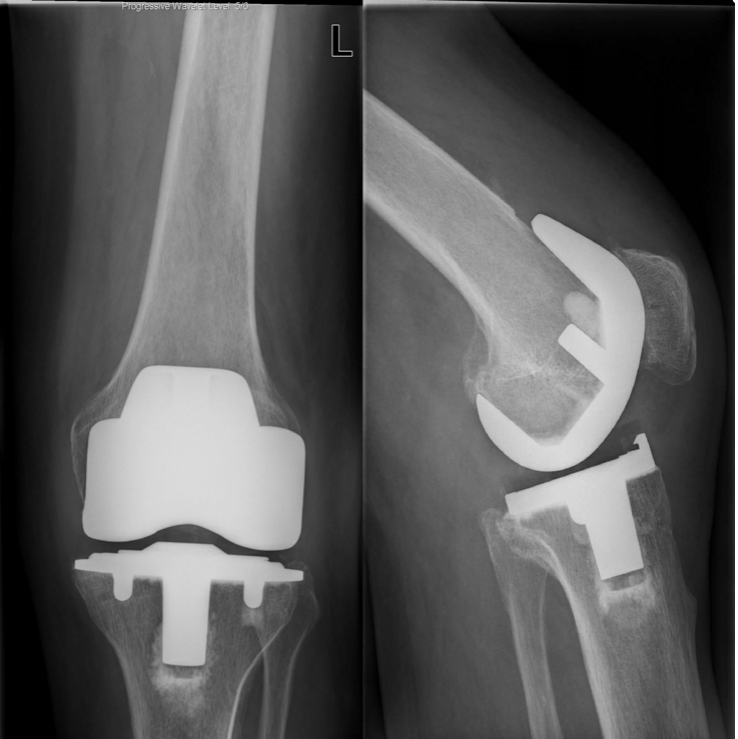
Radiographs of the prosthesis three years after total knee arthroplasty show normal alignment without evidence of loosening.

**Figure 2:**
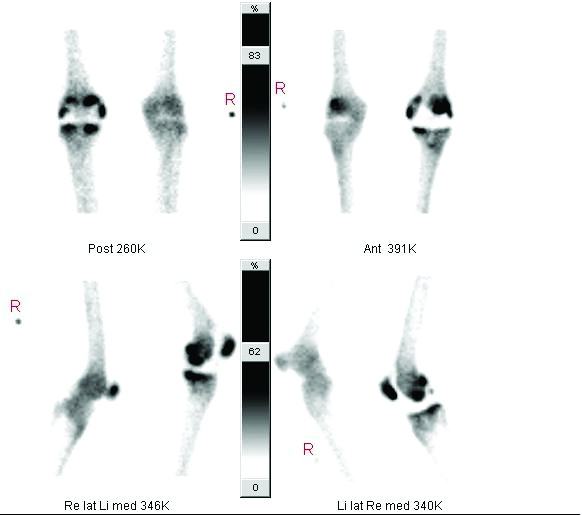
Triphasic bone scintigraphy shows an increased perfusion in the early phase and increased uptake in the static phase at the medial side of the femoral and tibial component and in the patella of the left knee.

A 63-year old male patient with a history of symptomatic osteoarthritis of the left knee underwent a Total Knee Arthroplasty (TKA) of posterior cruciate ligament retaining design (Kinemax, Stryker, Mahwah, New Jersey, USA) without a patella component. The postoperative course was uneventful. Three weeks later he presented to our out patient clinic with sudden swelling and discomfort of his left knee. Clinical examination demonstrated medial joint line tenderness and confirmed the patient's impression of joint effusion. Radiographs demonstrated a well-aligned TKA. All complaints, with exception of the knee effusion, declined progressively over a period of months. Two years postoperatively, the patient developed increasing pain and complained of "catching" of the knee. Physical examination showed a stable knee with a normal range of motion of 130 degrees flexion with no extension deficit. There was a moderate swelling and joint line tenderness medially. Standard radiographs showed a well-aligned TKA with no signs of loosening or polyethylene wear (Figure 1). Laboratory analysis including a complete blood count with differential, erythrocyte sedimentation rate, C-reactive protein and knee aspiration for cell count and culture excluded infection. A technetium 99m diphosphonate bone scintigraphy showed an increased perfusion in the early phase and increased uptake in the static phase at the medial side of the femoral and tibial component and in the patella of the left knee (Figure 2).

**Figure 3:**
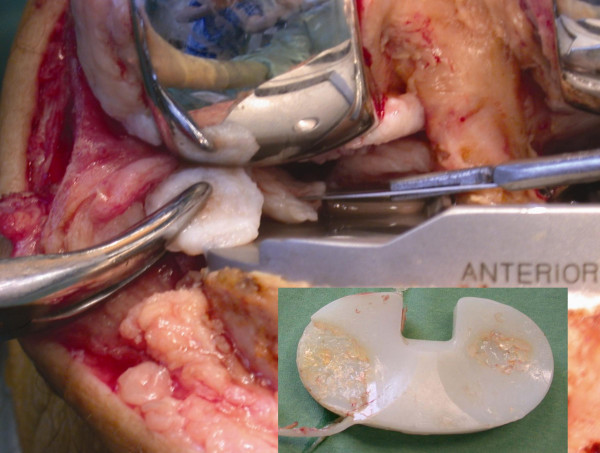
Photographs show a trapped posteromedial meniscus and severe damage of the polyethylene insert with polyethylene debris.

A diagnostic arthroscopy was performed to differentiate between a mechanical and a soft tissue related problem. Arthroscopy revealed a remnant of the posterior horn of the medial meniscus impinging between the posterior part of the femoral component and the polyethylene insert. There was also an important delamination of the anteromedial part of the insert with a crack at the ventral side associated with substantial synovitis and polyethylene debris scattered all around the joint. Slight delamination of the posterolateral part of the insert was visible. An arthrotomy showed no loosening of the femoral and tibial components of the TKA. There was no malrotation of both components. The tibial slope was not excessive (almost neutral). The trapped posteromedial meniscus remnant was removed (Figure 3) and a total synovectomy with an isolated exchange of the polyethylene insert was performed. Intraoperative cultures from both the fluid aspiration and the remnant meniscus yielded no micro-organisms. Postoperatively there were no complications with a complete resolution of all complaints and symptoms. At 3 years follow-up he remains complete symptom free with an unrestricted range of motion.

## Discussion

TKA is a successful procedure with a satisfactory outcome in patients with primary and secondary osteoarthritis of the knee. Unfortunately, a small group of patients complain about pain, recurrent knee effusion and limited range of motion postoperatively. Acute and low grade infection, "overstuffed knee", prosthetic loosening, rotational component malpositioning and flexion instabilities are the most recognised articular causes. Chronic synovitis from soft tissue impingement has rarely been reported [1-5]. Our patient had a remnant posterior horn of the medial meniscus trapped between the femur component and the polyethylene insert. This was responsible for the catching sensation of the knee and the recurrent pain. Because of the posteromedial impingement of the remnant meniscus, the contact stresses at the anteromedial side, and in lesser extend at the posterolateral side were probably higher and responsible for the severe polyethylene wear with delamination.

Since there was no real symptom free interval between the complaints and the index operation, we considered the impinging remnant meniscus a result of incomplete removal of the meniscus during the TKA procedure [2], rather than regenerated after surgical removal [1,3]. We consider the lack of a symptom free interval an important finding related to the impinging remnant meniscus in contrast to early polyethylene failure caused by other mechanisms. The value of bone scintigraphy in the diagnosis of prosthesis loosening is limited. Bone scintigraphy typically provides high sensitivity but exhibit variable specificity. An increased uptake can be seen many years after the implantation of TKA but the tracer uptake is generally mild or moderate and decreasing over time [6,7].

The diagnostic value of arthroscopy after TKA is controversial. It has been suggested that several complications of TKA, for example soft tissue-related problems, can successfully be managed by arthroscopy [4,9,10]. However, Van Mourik et al [8] stated that the indications for a diagnostic arthroscopy in painful TKA are, without any preoperative diagnosis, very limited. In our case, arthroscopy clearly highlighted the problem of localised polyethylene wear caused by a remnant meniscal part, warranting arthrotomy to perform a polyethylene insert replacement and resection of the remnant meniscus.

Early isolated polyethylene insert exchange in aseptic TKA is a rare procedure. Generally, most indications are associated with varied forms of soft tissue complications needing additional synovectomy, arthrolysis and ligament release. The lack of a symptom free interval may suggest an immediate postoperative problem caused by an impinging remnant meniscus. To our best knowledge, this is the first well-documented case reporting early polyethylene failure in TKA caused by an impinging remnant meniscus.

## Conclusion

Based on this report we emphasise on the importance of meticulous resection of the menisci during TKA and the diagnostic value of arthroscopy in unexplained pain and swelling after TKA with no signs of infection.

## Abbreviations

TKA – total knee arthroplasty

## Competing interests

The author(s) declare that they have no competing interests.

## Authors' contributions

RS conceived the study, participated in its design and coordination and helped to draft the manuscript.

BVR revised the article for intellectual content details.

CMF conducted the literature review and carried out the review of the patient's medical record in order to collect all the available information.

All the authors read and approved the final manuscript.
